# New Perspectives on Classical Alarmin Responses to Intestinal Helminth Infection

**DOI:** 10.1111/pim.70027

**Published:** 2025-09-12

**Authors:** Connor P. Lynch, Richard K. Grencis

**Affiliations:** ^1^ The Lydia Becker Institute of Immunology and Inflammation and the Manchester Cell‐Matrix Centre, Faculty of Biology, Medicine and Health University of Manchester Manchester UK

**Keywords:** alarmin, cell mediated immunity, helminth, mucosal immunity, parasite

## Abstract

Interleukins 33, 25, and thymic stromal lymphopoietin (TSLP) are core components of type two immune responses and have been studied extensively using helminth infection models. However, many questions remain regarding their cellular sources, their immune recipients, as well as how they shape immunity. Recent literature has demonstrated non‐epithelial alarmin production, acting primarily on lymphoid effector cells, and has suggested a role for alarmins in licensing of effector function in tissues during immunity, in dissent with conceptions of classical alarmins as epithelium‐derived, myeloid‐targeting, and induced prior to adaptive responses. This review examines recent findings in alarmin helminth interactions at barrier sites and discusses the wider implications for how alarmin responses are conceptualised.

## 1 Helminths

Intestinal helminths are a class of phylogenetically diverse, multicellular eukaryotic parasites which colonise the gastrointestinal tract during their life cycles. Hundreds of intestinal helminth species exist in the wild and are used experimentally across laboratories worldwide, but immunology researchers generally focus on three major mouse models: the hookworm *Nippostrongylus brasiliensis* (*Nb*), the roundworm *Heligmosomoides polygyrus bakeri* (*Hp*), and the whipworm *Trichuris muris* (*Tm*). Two key features of anti‐helminth immune responses are: (a) the involvement of the classical alarmins interleukin (IL)‐33, IL‐25, and thymic stromal lymphopoietin (TSLP), and (b) the production of IL‐13 and other type two cytokines by lymphoid cells (type two innate lymphoid cells (ILC2s) or type two effector CD4+ T cells (TH2 cells)) to effect parasite expulsion [1, 2]. Fundamentally, the unifying conditions necessary for the initiation of type two adaptive immune responses remain unclear, as discussed in a recent review [3], and parasite infections remain indispensable immunological research tools, being major drivers of the evolution of type two immunity. Expression of IL‐13 receptor components in the intestine is necessary for the epithelial responses necessary with parasite expulsion [4, 5], and experiments with gut epithelial organoids indicate that IL‐13 likely mediates these effects via epithelial cells directly [6]. The contribution of indirect IL‐13 stimulation of epithelial responses via, for example, sub‐epithelial fibroblasts remains to be further investigated.

Life cycles determine which host cells and tissues encounter parasites and thus are a key determinant of how immunity develops to expel them. *Nb* exhibits a complex life cycle, in which infectious larvae penetrate the skin, circulate to the lungs via the blood, and enter the small intestine by being coughed up and swallowed, becoming egg‐producing adults in the small intestine. Expulsion in mice usually occurs between 4 and 7 days p.i., and most mouse strains are highly resistant to *Nb* infection, with chronic infection being rarely established in wild type strains. *Nb's* multi‐tissue life cycle, however, provides an important tool for investigating cross‐tissue type two immunity [7, 8]. While tuft cell detection of the parasite in the small intestine, leading to IL‐25 release and ILC2 production of effector cytokine IL‐13, remains the key mechanism in expulsion, recent studies have uncovered roles for immune cells at the skin and lung in containing parasites or seeding other tissues with activated immune cells [7], questions remain regarding the contribution of alarmins at these earlier barrier sites, discussed later.

Ingested *Hp* larvae penetrate the small intestinal submucosa, and moult twice before re‐emerging into the lumen, coiling around villi and mating to produce eggs in faeces, which hatch and develop in the environment into infectious L3 larvae. Primary *Hp* infections are not readily expelled from many inbred strains of mouse, including C57BL/6 mice, likely due to extensive immunomodulation by the parasite targeting IL‐33, its receptor ST2, and IL‐13 [9, 10, 11, 12]. Researchers often experimentally clear primary *Hp* infections with anthelminthics, prior to the administration of secondary infections, which are rapidly expelled via type two immune responses [13]. Much *Hp* research focuses on this immunomodulation and how it impacts other immune responses, in models such as parasite co‐infection [8, 14, 15]. Although expulsion does not occur in mouse models, mucus and antimicrobial peptide production by goblet cells has been associated with increased expulsion of the parasite [16]. The formation of granulomas in the lamina propria has been studied as a mechanism of parasite destruction. This is evidenced to be co‐ordinated by macrophages and granulocytes following IL‐4 and IL‐13 signals from TH2 cells [17, 18]. Both granuloma and epithelial response efficacy in repelling parasite infection appear to be limited to secondary infection, but more modern analysis using conditional knockouts, imaging, and in vitro models would permit better understanding of these mechanisms.

The *Tm* life cycle begins when embryonated eggs are ingested, which hatch in the caecum upon exposure to characterised microbiome components [19, 20, 21, 22], after which larvae embed into epithelial cells by approximately 90 min p.i., [23]. In this niche, the synctial tunnel, larvae undergo four moults, mating as adults from around 28 days p.i., and producing eggs into the lumen which embryonate in the environment following defecation. *Tm* immunity is dose dependent: low egg doses (LD, ~20 eggs) generally result in TH1‐mediated adaptive responses and chronic infection, while high doses (HD, > 100 eggs) infection results in TH2 responses including IL‐13 production, the key cytokine effecting stroma‐mediated expulsion [24, 25]. This dose‐dependent immunity allows manipulation of host immune responses to *Tm* which can be accessed via parasite burden. *Tm* infection presents a more tractable model of type two immunity induction than either *Nb* or *Hp* (reviewed in [26]), as the same parasite can be used to induce both type one and type two immune responses, making it an ideal model for examination of the basic conditions necessary to induce type two immunity, a significant question and challenge in the field [3]. While it appears likely that low doses of infection with other parasites may alter immunity, acute infection being almost universal in *Nb*‐infected mouse strains and rare during primary *Hp* infection makes alteration of dosage to investigate immunity less useful in these models. The key expulsion mechanism for Tm is IL‐13‐mediated increases in epithelial cell turnover [24], as well as increases in and changes to mucus production by goblet cells [27].

## 2 Alarmins

No official definition exists for what constitutes an alarmin, but they are considered to encapsulate secreted proteins which are rapidly produced by non‐immune cells in response to damage and/or detection of infection, usually at barrier sites, for the purpose of commencing immune responses. This separates alarmins from other cytokines produced by immune cells which polarise immunity (e.g., IL‐4) or stimulate pathogen destruction or expulsion directly (e.g., IL‐13), as well as damage‐associated molecule patterns (DAMPs) which perform normal cellular functions and secondarily function as damage indicators when released from damaged or dying cells (e.g., DNA). However, despite being broadly conceptualised as being initiators of immunity, evidence for alarmin activation of effector lymphoid cells is far more prevalent than evidence suggesting classical alarmins to act on antigen presenting cells. A 2016 study by Van Dycken et al. [28] demonstrated that triple alarmin receptor KO mice (lacking TSLPR chain CRLF2, ST2 chain IL‐1RL1, and IL‐25R chain IL‐17RB) exhibited normal lymph node induction of T cells during *Nb* infection, but severely impaired ILC2 numbers and TH2 effector responses in the lung, consistent with a role for alarmins in enabling effector functions of T cells and ILCs rather than signalling APCs. Poor effector responses but normal adaptive priming in the lymph node in IL‐33 knockout mice during *Nb* infection has been suggested by an earlier study [2], which also demonstrated increased *Nb* burden in the lung during secondary infection, and a role for IL‐33 in promoting parasite expulsion, but not in promoting T cell production of IL‐4 in the lymph node. The paucity of in vivo evidence for expression of functional alarmin receptor expression on myeloid cells, in contrast to well‐documented expression on lymphoid cells [28, 29, 30, 31, 32, 33, 34, 35, 36, 37], speaks against a role for alarmins polarising APCs. The importance of lymphoid cells to alarmin‐mediated immunity considered, they will be the focus of this review, but granulocytes [38, 39, 40] myeloid cells [41, 42], and B cells [43, 44] have all been shown to contribute to worm expulsion, although the mechanisms at play often remain unclear. This review will also focus on mouse studies in which causal immune relationships are discerned: the level of functional conservation between mouse and human alarmin biology is unclear.

Although often considered to act similarly, the three classical alarmins play distinct immunological roles. A study from Gurram et al. [45], dissecting responses to OVA immunisation and papain challenge in the lung, found that while ILC2s and IL‐33 were essential to driving the eosinophilia associated with papain responses, OVA immunisation‐provoked eosinophilia was, instead, dependent on TSLP and TH2 cells. The association of TSLP with T cells during OVA responses, contrasting with IL‐25 and IL‐33 eliciting ILC2 responses during papain challenge, has been previously reported [34]. Functionally, TSLPR knockout mice demonstrate robust protection from eosinophilic oesophagitis, not observed in knockouts for IL‐33 receptor ST2 [46], demonstrating that eosinophilia produced by the two alarmins exhibits clinically important differences. A study of basophil and TSLP responses during HD *Tm* infection has characterised distinct contributions by alarmins to circulating granulocyte makeup, with TSLP promoting basophilia; IL‐25, mastocytosis; and IL‐33, eosinophilia [39], suggesting differing effects on the bone marrow and the posture of the immune system. Alarmin and alarmin receptor knockout mice also exhibit varying susceptibilities to various helminths, as later discussed. These differences are likely the result of differential expression of functional receptors for alarmins on immune cells, combined with alarmins being produced by distinct cells following distinct stimuli. In general, the characterisation of alarmin sources has been held back by a lack of effective reagents, with characterisation of their sources being determined largely via reporter mice, developed for IL‐33 in 2012 [47], IL‐25 in 2016 [32], and TSLP in 2015 [48]. However, recent research has created a new and more complex picture of alarmin sources across tissues and challenges, which calls into question the idea of alarmins as primarily epithelial products, excepting interleukin 25.

## 3 Interleukin‐25 Biology

A member of the IL‐17 family of inflammatory cytokines, IL‐25 was first connected with helminths in a 2006 study showing that a type two cytokine‐producing, non‐B non‐T cell population in the intestine, reliant on IL‐25 signalling, promoted *Nb* expulsion [49]. IL‐13 production by these “nuocytes”, now referred to as ILC2s, induced by IL‐25 with partial compensation from IL‐33, was observed as the key driver of *Nb* expulsion [50]. The sole source of IL‐25 in the small intestine was later determined to be an epithelial cell type known as tuft cells [32]. A study examining the impact of classical alarmin receptor knockouts on ILC2s across the body indicated that while *Crlf2* (encoding the specific TSLPR chain) was expressed evenly across examined tissues, ST2 receptor transcript (encoding the IL‐33 receptor chain) expression was strongest in the lung and adipose tissue, while *Il17rb* (encoding the specific IL‐25R chain) was more strongly expressed in the small intestine than any other alarmin receptor, and expressed minimally elsewhere [29]. Although the specific affinities of the receptors, as well as expression of their shared chains, limit definite conclusions on the level of alarmin signalling in each tissue, this expression pattern is consistent with IL‐25 being predominantly a small intestinal alarmin, and acting largely via the stimulation of ILC2s, rather than TH2 cells. Research from the von Moltke lab and others indicated that the lung contains chemosensory leukotriene‐producing brush cells, functionally analogous to intestinal tuft cells [51], which produce increased IL‐25 during various allergic challenges, similar to their intestinal counterparts [52]. Although leukotriene production in the lung has been demonstrated to contribute to ILC2 responses to *Nb* alongside IL‐33 [53], IL‐25 does not appear to contribute to *Nb* immunity during the lung phase [54], owing likely to a dearth of tuft cells in the distal lung through which the parasite migrates. One study observed *Trichinella spiralis* (*Ts*) infection was able to increase IL‐13 production by ILC2s in the lung, resulting in increased parasite trapping as the new boren larvae move through the circulation to the muscles [7], although whether the IL‐13 production by ILC2s is driven by IL‐25 or another alarmin was not determined, and IL‐33 has been observed to act redundantly with IL‐25 in the lung to support ILC2 responses [45].

Regarding the binding of IL‐25 to its receptor, while it has long been known that both IL‐17Rα and β chains are necessary for signal transduction [55], another role for IL‐17Rβ has been recently characterised in tuft cells themselves: reporter mice have indicated that intestinal IL‐25 is expressed constitutively [32], and a recent pre‐print indicates that the expression of IL‐17Rβ by tuft cells allows sequestration of the protein, which is released, rather than only transcriptionally upregulated, by succinate stimulation [56]. Current research on tuft cells has focused on the intracellular signalling pathways which connect parasite detection to effector responses: two simultaneously published papers in Immunity (2024) describe both luminal secretion of acetylcholine to impair parasite function [57] and basal secretion to increase chloride secretion by enterocytes [58], the papers concurring that neither mechanism relates directly to IL‐25. This research does however highlight the importance of identifying key producer cells in the study of alarmins: mechanisms behind alarmin upregulation can only be investigated in detail once such cells have been identified.

Recent work emphasising the role of neurons in regulating intestinal responses has indicated that ILC2s require neuronal product NmU23 to produce effector cytokines in vitro, which could not be induced by IL‐25 or IL‐33 alone [59]. While one study has indicated that NMUR1 receptor expression on ILC2s, necessary for the detection of neuron‐produced NMU, can be increased by IL‐25 treatment [60], other labs have found IL‐25 and IL‐33 to stimulate IL‐13 production by ILC2s without neuronal molecules [61]: the extent to which IL‐25 requires neuronal mediators for effective activation of ILC2s, and the input of neurons into type two immunity more broadly, remains unclear and is an area of great interest to mucosal immunologists at present.

## 4 IL‐25 and *N. brasiliensis*

Three 2016 papers established the mechanism through which IL‐25 influences helminth immunity: A study from Gerbe et al. [62] examined tuft cells, chemosensory intestinal epithelial cells, during *Nb* infection: tuft cell knockout mice exhibited an inability to clear the parasite by day 42 p.i. and poor IL‐13 production in the intestine, and observed production of IL‐25 by tuft cells in infected mice. Howitt et al. [63] observed the expansion of tuft cells during protist and *Hp* infections as well as tuft cell chemosensation of succinate, proposed as an initiator of tuft cell expansion and IL‐25 production, with knockouts for chemoreceptor TRPM5 shown to impair goblet cell responses and eosinophilia in response to protist infection. Thirdly, von Moltke et al. [32] used an IL‐25 reporter mouse to demonstrate tuft cells as the sole intestinal IL‐25 source, IL‐25 as necessary for *Nb* expulsion, and showed that in addition to inducing IL‐13 production by ILC2s via IL‐25, IL‐13 further upregulated tuft cell differentiation from epithelial stem cells, establishing a positive feedback loop. This remains understood as the central mechanism by which IL‐25 contributes to intestinal helminth immunity. Importantly, von Moltke also noted that CD4‐conditional IL‐13 knockout did not significantly impact tuft cell expansion, suggesting ILC2s as the key IL‐13 producers and expulsion effectors, with little role for TH2 cells during primary *Nb* infection [32]. A recent study from Gurram et al. noted increased susceptibility to primary *Nb* infection in TH2‐deficient (h*CD2* ^Cre^ *Gata*3^fl/fl^) mice [45], but similarly concluded that this was the result of decreased ILC2 production of IL‐13 due to impaired TH2‐ILC2 crosstalk, suggestive against IL‐25 signalling of TH2 cells. The paper further observes that the blockade of IL‐25 and IL‐33 together, but neither alarmin separately, decreased ILC2 expansion in the lungs during papain challenge, indicating a degree of redundancy between IL‐33 and IL‐25 in supporting ILC2 responses. However, in the context of *Nb* infection, tuft cell hyperplasia in the lung has been observed not to occur [7]: further research is necessary to describe mechanisms for alarmin contribution to *Nb* infection out with IL‐25 in the intestine. Earlier examples of IL‐25 supporting effective TH2 activity, such as enabling T cell IL‐9 production during *T. spiralis* infection [64], may in fact be via ILC2 activation, but more detailed study of infections, in the vein of Gurram et al.'s study, would be required to uncover the alarmin‐cell interactions during specific infections.

## 5 IL‐25 and *H. polygyrus*

Although best characterised during *Nb infection*, IL‐25 also contributes to *Hp* immunity: weaker tuft cell expansion during *Hp* infection, compared to *Nb* or *Ts*, has been recorded, potentially due to the sub‐epithelial localisation of the parasite during early infection preventing effective chemosensation by tuft cells. *Hp* extensively inhibits IL‐33 activity [9, 10, 65], and the normally chronic course of *Hp* infection in mice suggests against effective activation of the tuft cell‐IL25‐ILC2 circuit which expels *Nb* infection. However, an increase in *Il25* transcription during secondary *Hp* infection, noted to decrease parasite fecundity, has been observed [33]. This is suggestive of a role for IL‐25 in directly or indirectly enabling memory T cell function during secondary *Hp* infection, demonstrated to be necessary for expulsion in experiments with SCID and nu/nu athymic mice which lack T cells [66], although whether T cells rather than ILC2s dominate IL‐13 production during *Hp* infection, or whether they are enabling ILC2 production of the cytokine, has not been determined. A study in IL‐17RB knockout mice has shown increased faecal egg counts at days 14 and 18 post‐primary *Hp* infection, and impaired worm clearance following secondary *Hp* infection [33], suggesting a role for IL‐25 signalling to ILC2s in containing primary *Hp* infections and expelling secondary infections. As mentioned, previous studies have noted an increase in mast cells in IL‐25 treated mice [39], and a study examining mast cells observed higher worm counts at d21 p.i. in KitW/KitW‐v mice, which lack mast cells, as well as increased susceptibility to *Hp* [67]. However, they also observed a complete ablation of all increased alarmin transcription in the small intestine in these mice during infection, suggesting a defective intestinal environment which may go beyond selective mast cell deficiency. Questions remain regarding the degree of chemosensation of *Hp* by tuft cells, and the relative contribution of ILC2s and TH2 cells to expulsion of the parasite.

## 6 IL‐25 and *T. muris*

Evidence for IL‐25's contribution to immunity to *Tm*, which inhabits the caecum, a tuft cell‐deficient environment relative to the small intestine [68], is less substantial. During trickle *Tm* infection, in which repeated low doses are administered to mimic natural infection, minimal expansion of tuft cells was observed in the caecum [69], with additional data from the author showing no significant increase in tuft cells at day 19 p.i. in either LD or HD infection regimens [68]. One study has observed susceptibility to HD Tm infection in IL‐25 knockout mice [70]: a subsequent study has replicated this finding, and observed a reversal of susceptibility when IL‐25‐elicited progenitor immune cells were transfused into knockout mice [71]. It should be noted, however, that *Tm* expulsion normally occurs between days 19 and 25 [72], meaning parasite counts at d20 may indicate delayed expulsion rather than chronicity, and worms were incompletely cleared in IL‐25 KO mice from both treated and untreated groups, suggesting only a partial ability for IL‐25‐stimulated immune cells to enact *Tm* expulsion. Neither study observed upregulation of IL‐25, expansion of tuft cells, or increased ILC2 counts in the caecum during HD infection.

Exogenous IL‐25 administration has, however, been shown to render normally susceptible AKR mice resistant to HD *Tm* infection [70], but not SCID mice lacking T and B cells. Recent research has also uncovered a role for ILCs in contributing to HD *Tm* clearance via Areg production [73], and with CD4+ T cells [74, 75] and IL‐4 [76] being indispensable to the IL‐13 production which enables expulsion. It appears likely that ILC2‐TH2 crosstalk would permit exogenous IL‐25 to improve *Tm* expulsion out with the tuft cell circuit: a study examining *Hp* infection observed that Areg signalling (in this model, autocrine) was required to enable TH2 cell production of IL‐13 [77]. The relative contribution of TH2 cells and ILC2s to IL‐13 production during HD Tm infection remains to be determined, and although TH2 cells remain the definitive effector cell in expulsion of HD *Tm* infection, at least in C57BL/6 mice, a role for ILC2s in enabling T cell effector responses continues to be developed.

Overall, IL‐25 is arguably the best characterised of the alarmins, long identified as being produced by epithelial tuft cells in the small intestine following chemosensation of parasite infection, and acting primarily on ILC2s to induce rapid production of effector cytokines, primarily IL‐13. New research in the field, building on earlier findings, has identified how IL‐25 is retained in tuft cells and released unconventionally following stimulus [56]. Its level of direct interaction with TH2 cells during helminth infection is not clear, but considering ILC2 release of Areg is a topic of present interest [73], indirect signalling of TH2 cells via IL‐25 suggests itself as an interesting mechanistic axis in type two immunity. Evidence is robust that IL‐25 contributes to expulsion of *Nb* via ILC2s, with a role to play in Hp immunity, complicated by the chronic course of infection. *Tm* immunity, while not seemingly dependent on IL‐25 during HD infection, presents itself as a useful model of ILC2‐TH2 interactions via the examination of the role of IL‐25, although expulsion appears primarily driven by other alarmins.

## 7 Interleukin‐33 Biology

IL‐33, a member of the IL‐1 family of inflammatory cytokines, is unusual in a number of ways: the uncleaved IL‐33 protein contains, in addition to its C‐terminal domain which binds to the ST2 receptor to mediate IL‐33's effects on cells, an N‐terminal domain which binds to chromatin, and a central activation/cleavage domain which connects the two, tethering the cytokine domain of IL‐33 to the nucleus of the producer cell [78]. This means that, like IL‐25, IL‐33 translation does not result in immediate cytokine release, complicating measurement of its activity. IL‐33 can be released during forms of unregulated/inflammatory cell death, allowing the C‐terminus to bind ST2 and provoke strong inflammatory responses in immune cells. Genetic deletion of the N‐terminal domain, resulting in uncontrolled IL‐33 release from cells, results in multi‐organ eosinophilia and death in mice [79]. Upon being released, the activation domain can be cleaved by various immune cell‐produced proteases to increase activity, possibly through higher affinity binding to ST2 [80, 81]. During non‐inflammatory forms of cell death, the C‐terminal, cytokine domain is cleaved intracellularly by caspases 3 and 7 to prevent inflammation [82], further complicating examinations of IL‐33 protein activity. Several recent papers have described live cell secretion of IL‐33 via upregulation of Gasdermin pores, accompanied by cleavage of full‐length IL‐33 from the N‐terminal domain [83], although the intracellular proteases necessary to cleave IL‐33 from the nucleus to permit transit through Gasdermin pores have not been characterised.

## 8 IL‐33 Sources

Unlike IL‐25, the complicating factor in the study of IL‐33 sources is the breadth of its expression: a study characterising IL‐33 reporter mice showed expression in epithelial cells from the lung, skin, stomach, and vagina; retinal glial cells; vasculature endothelial cells of the colon during DSS‐induced colitis; and fibroblastic reticular cells in various lymphoid tissues [47]. A recent study examining fungal symbionts in the small intestine observed expression of *Il33* only by epithelial cells [84], another 2024 study observing increased *Il33* in the bases of crypts in the small intestine treated with DSS [85]. Single cell sequencing analysis of the murine colon indicated both fibroblasts and epithelial cells as substantial *Il33* expressors [86]. Untangling the relative contribution of these various sources to helminth immunity is complicated by this broad expression and IL‐33's complex release mechanisms. Given IL‐33's capacity to stimulate eosinophilia [39, 45], the eosinophilia enrichment of the jejunum over the duodenum and ileum suggests higher basal IL‐33 signalling potential in specific regions of the intestine, outlined by a study examining eosinophils as regulators of intestinal morphology at homeostasis in an IL‐33‐dependent way [87]. Although IL‐33 expression in this paper is observed in both epithelial and lamina propria‐resident cells, the relative contribution of these cell types to immunity remains contentious, as discussed later.

ILC2s and TH2 cell numbers are expanded in the lung during *Nb* infection, and this expansion was impaired by IL‐33 knockout in stromal cells [88], although whether IL‐33 directly engages TH2 cells in this model is unclear. IL‐33 knockout mice have been noted to poorly induce ILC2 and TH2 IL‐13 production during secondary *Nb*, and exhibit higher d6 p.i. parasite burdens, but induce IL‐4 and IgE in the lymph node comparably to infected wild types [2], consistent with the theorised alarmin function in effector cell licensing in tissues rather than signalling APCs. This could, however, also be the result of poor ILC2 activation resulting in improper TH2 activity following migration to tissues, as discussed earlier [73, 77].

A previously mentioned study observed that treatment of neuronal organoids with IL‐33 produced increased NMU secretion [59], a compound demonstrated in vivo to stimulate cytokine ILC2 cytokine activity [60], suggesting that IL‐33 may impact ILC2s and TH2 cells in vivo indirectly via neurons [59]. Interestingly, one paper has shown that treatment of ST2+ ILC2s with IL‐33 produced an IL‐25‐responsive population of ILC2s [89], suggestive that IL‐33 may act upstream of IL‐25 to licence lymphoid cells for IL‐25‐mediated IL‐13 production. IL‐33 has been shown to be unable to induce IL‐13 secretion by TH2 cells without stimulation via Areg [77], consistent with this licensing role for the alarmin in lymphocytes. Inversely, IL‐33 has been observed to directly increase expression of the α7nAChR receptor (a receptor for various neurotransmitters) on ILC2s and reduce their cytokine production, indicating that IL‐33 may both act on ILC2s via neural signals and *vice versa* and is capable of both increasing or decreasing ILC2 activity, depending on local neuron activity [90] and the balancing signals of other cytokines.

## 9 IL‐33 and *N. brasiliensis*

The primacy of the IL‐25 response circuit to *Nb* immunity has largely rendered questions around IL‐33 responses to the parasite secondary, but interesting research has emerged drawing into focus some points of contention in IL‐33 research, using the *Nb* infection model. One study has observed that small intestine epithelium‐conditional IL‐33 KO mice exhibit impaired clearance of *Nb* infection from the gut, while dendritic cell‐conditional (CD11c‐Cre) knockouts of IL‐33 produce stronger type two immunity and parasite clearance [91]. The study also noted constitutive cytoplasmic localisation of IL‐33 in DCs, in contrast to nuclear localisation of IL‐33 in epithelial cells, and characterised dendritic cell IL‐33 as likely released through perforin‐2 pores to stimulate regulatory T cells [91]. Cytoplasmic localisation is likely to occur at least transiently in some cell types, given the various instances reported of live cell secretion of the protein via Gasdermins. Administration of the *Hp*‐derived IL‐33 inhibitor HpARI has been observed to increase susceptibility to *Nb* in the intestine at d6 p.i., [9]. This could be due to parasite retention in the lung, where reduced eosinophilia and tissue remodelling was observed, and where IL‐25 seemingly does not contribute to immunity [54], rather than impaired immunity at the intestinal stage. Data from Gurram et al. [45] showed ILC2 responses to the papain‐challenged lung to be largely ST2 dependent in this model, but that ST2 knockout on ILC2s only partially impaired tuft cell expansion and parasite clearance during *Nb* infection. Mucin responses in the lung during Nb infection also appear unimpacted in ST2 knockout mice [7], suggesting minimal IL‐33 signalling. In the intestine, ST2 has been observed as less robustly expressed on ILC2s than at other barrier sites: ILC2‐conditional ST2 knockout mice in one study experienced partial loss of ILC2 numbers in the small intestine, less substantial than that observed in the lung, adipose tissue, or mLN [30], supporting that IL‐33 may contribute to immunity to *Nb* at the lung phase of infection. However, IL‐33 knockout mice were observed in another study to exhibit impaired ILC2 activation in mesenteric lymph nodes, in addition to increased *Nb* small intestinal burdens [92], suggestive of IL‐33 influencing ILC2 activity directly in the intestine. No decrease in ILC2 IL‐13 production was noted, but in light of earlier discussed literature indicating ILC2s as potential enablers of TH2 functionality, IL‐33 likely plays a non‐redundant role in the expulsion of *Nb*, with contributions at the lung and intestinal stages of infection, and impacts on ILC2s and T cells still to be fully explored.

## 10 IL‐33 and *H. polygyrus*

The helminth most closely associated with IL‐33 responses is *Hp*, which produces molecules to re‐tether IL‐33 to DNA, but the susceptibility of most mouse strains to infection, perhaps due to the efficacy of IL‐33 inhibition, means a clear role for IL‐33 in promoting expulsion of *Hp* has been until recently challenging to demonstrate. Parasite factor HpARI has been shown to impair ILC2 responses during *Alternaria* challenge to the lung, as has ST2‐binding HpBARI [9]. A recent study has, however, demonstrated that, following treatment with isolated anti‐HpARI IgG2 from previously infected animals, mice develop TH2 and ILC2 responses and control parasite egg release more effectively [65], indicating that without immunosuppression, IL‐33 may be able to promote *Hp* expulsion. Parasite expulsion was in this study only moderately improved, potentially due to the breadth of immunomodulators produced by *Hp*, including TGFβ mimics [11] and IL‐13 inhibiting molecules [12]. One study measured 40‐fold increases in *Il33* in small intestinal homogenate at d2 post‐*Hp* infection, which increased to 150‐fold at d4 before returning to 40‐fold at d6 [67]. The location of the parasites in the lamina propria at these timepoints suggests fibroblasts as sources of IL‐33 in this context, particularly considering that stromal expression of IL‐33 has been documented in the large intestine [86, 87]. Given that primary *Hp* infections are not readily expelled by mice, that IgG responses appear key to expulsion during secondary infection [93], and that TH2 cells generated during Hp infection are capable of expelling Nb infection in the lungs in an IL‐33‐dependent manner, IL‐33's input into adaptive memory cell formation may contribute more to immunity than its direct stimulation of effector lymphoid cell cytokine activity in the *Hp*‐infected small intestine. The work suggesting epithelial and myeloid IL‐33 as mediating susceptibility and resistance, respectively, during *Nb* infection remains to be explored during *Hp* infection.

## 11 IL‐33 and *T. muris*

As for IL‐25, administration of recombinant IL‐33 has been demonstrated to generate resistance to HD *Tm* in normally susceptible AKR mice, and an increase in *Il33* transcription in the caecum (20‐fold over naïve at d3) has been measured in resistant BALB/c mice [94]. Immunity could not be induced by IL‐33 administration in SCID mice lacking T and B cells, affirming that T cells rather than ILC2s are the likely drivers of immunity during HD *Tm* infection, as noted in IL‐25 administration [70]. Additionally, high‐fat diet appears to promote expulsion of HD *Tm* via increased IL‐33 signalling of T cells [95]. However, IL‐33 KO mice in this study do not appear to be susceptible to HD *Tm*, suggesting IL‐33 does not drive immunity during unmodulated HD *Tm* infection. Co‐infection with *Hp* prior to HD *Tm* administration has been demonstrated to result in susceptibility to the latter, despite normal TH2 signatures in the draining lymph node [96]. This would be consistent with *Hp*'s demonstrated capacity for suppressing IL‐13 function in producing epithelial responses [12], rather than due to IL‐33 inhibition. As mentioned previously, this is consistent with a role for IL‐33 in licensing lymphoid effector function in tissue, rather than being involved in APC priming.

Overall, IL‐33's present contribution to all three major helminth infection models is complex: the respective pro‐ and counter‐TH2 influence of epithelial and myeloid IL‐33 in *Nb* infection, and the dominance of the IL‐25 circuit in promoting expulsion complicates an assessment of IL‐33's overall contribution. Recent work in neutralising *Hp*s immunomodulatory secretions suggests that IL‐33 is capable of promoting expulsion, although whether through ILC2s, T cells, or indeed macrophages or eosinophilia remains unclear at this time. IL‐33 appears capable of promoting immunity to HD *Tm*, possibly by stimulating TH2 effector function in tissues; however, the cytokine is ultimately dispensable to normal immunity in C57BL/6 mice. In all cases, the sources of the alarmin have not been determined conclusively, but lymphoid cells have been identified clearly as recipient cells, with a role for myeloid ST2 signalling yet undemonstrated.

## 12 Thymic Stromal Lymphopoietin Biology

A cytokine from the lymphocyte development‐associated IL‐7 family, TSLP was initially characterised as a non‐IL‐7 lymphocyte survival factor produced by a thymic stromal cell line [97]. It was investigated as a lymphocyte‐stimulating developmental cytokine in the 1990s and early 2000s [98, 99, 100, 101] due to its connection with T cell growth factor IL‐7: the TSLP receptor is composed of the unique CRLF2 chain and the shared IL‐7Rα chain, and TSLP likely arose from IL‐7 during a gene duplication event [102]. Early studies in mice initially suggested TSLP as only stimulating lymphocytes [100, 101, 103], from which CRLF2 receptor chain was also initially cloned [104]. Interest in the impact of murine TSLP on myeloid cells followed several studies in which human TSLP was observed to promote type two inflammation in human myeloid cells in vitro [105, 106] and in vivo [107]. Given the numerous studies identifying TSLP as influencing myeloid cells in humas [105, 106, 107, 108], this may represent a substantial difference between human and murine alarmin biology, but more evidence is required to definitively exclude a role for TSLP in influencing murine immunity via myeloid cells. An influential 2005 study posited that in mice, too, TSLP could activate bone marrow‐derived DCs [109], although observed expression of the IL‐7Rα on DCs was poor, and increases in observed indicators of activation following TSLP treatment were minor. This study also ignited interest in TSLP's role in asthma, noting TSLP overexpression in lung epithelial cells to induce an asthmatic phenotype, resulting eventually in the development of Tezepelumab for treatment of human asthma [110]. Following this paper, murine TSLP has been conceptualised as being epithelium‐derived and influencing immunity via DC activation, although murine DC responsiveness to TSLP has been little characterised: a 2017 study has demonstrated that IL‐4‐induced IL‐7Ra expression by splenic DCs was a prerequisite for responsiveness to TSLP, and found increased CD80 expression and phosphorylation of STAT5 (through which ligated TSLPR signals via JAK2 [111]) on IL‐4‐treated DCs stimulated with TSLP [112]. DCs are thus capable of responding to TSLP in vitro, but TSLP signalling of DCs in vivo has not yet been demonstrated. A number of studies have documented murine ILC [113] and T cell [36, 37, 114] expression of the TSLPR in vivo and responsiveness to TSLP, without identifying TSLP producer cells, consistent with early literature noting numerous effects of TSLP on lymphoid cells and strong expression of the receptor on TH2 cells in particular.

## 13 TSLP Sources

The sources of TSLP have eluded characterisation likely due to a lack of effective reagents, such as antibodies for staining, and poor representation in single cell sequencing databases: only 19 *Tslp* + cluster entries exist on the Panglao single cell aggregator, compared with 243 for IL‐33. Although a TSLP reporter mouse was developed in 2015, robust expression of *Tslp* was detected only in thymic epithelial cells and keratinocytes, with minor expression by dendritic cells and basophils also noted [48], while the lung and gut epithelium were not examined in the initial paper or any subsequent study. A second reporter mouse developed in 2024 observed fibroblast, but seemingly not epithelial [115], expression in the small intestine at homeostasis, calling into question the basis for epithelial expression of the protein at non‐skin barrier sites. A recent study has indicated that macrophages, rather than epithelial cells/keratinocytes, are the sole expressors of *Tslp* during infection with the protozoan parasite *Leishmania*, supporting ILC2 responses [116]. Notably, IL‐33 and IL‐25 knockout mice did not exhibit the same defects as TSLP knockouts in ILC2 activation, suggesting minimal redundancy between alarmins in this setting. Keratinocyte production of TSLP in the skin has been observed by numerous labs during inflammatory challenge [115]: whether keratinocytes or macrophages produce TSLP in the skin during helminth infection, such as *N. brasiliensis* , has not been demonstrated, and TSLPR knockout appears to have minimal impact on the course of *Nb* infection [117].

Despite the key role of TSLP in early asthma mouse modelling, TSLP cellular sources during challenges to the lung are not well characterised, although one study has documented TSLP production by lung adventitial stromal cells, capable of supporting ILC2 responses [88]. A recent human study of asthma biopsies observed minimal indication of *Tslp* expression by epithelial cells [118], and use of Nb infection as an inducer of type two immunity in the lung may permit more mechanistic examination of TSLP activity at this site.

Another recent study has indicated *Tslp* expression in telocytes, fibroblasts underlying the intestinal epithelial layer, after feeding using a reporter mouse, and measured subsequent TSLP‐dependent expansion of ILC2s [115]. ILC2 responses were only moderately impacted by TSLPR KO, and feeding‐induced increases in TSLP expression were modest in this model, but a combination of staining and sequencing strongly supported contributions by fibroblasts to TSLP production in the small intestine. The study also showed little to no expression of TSLP by small intestine epithelial cells via in situ hybridisation. Immunofluorescent staining has previously suggested epithelial production of TSLP in the caecum and large intestine [119], and subsequent papers presume epithelial restriction of the alarmin [120], but further data supporting this conclusion is limited. Recent work has characterised small intestinal tuft cells as being capable of producing TSLP when transcription factor Spi‐B is knocked out, and that this TSLP can play a functional role in both Hp immunity and food allergy [121]. They observe minimal tuft cell *Tslp* expression in wild type mice, and a previous study observed essentially no non‐tuft cell *Tslp* expression in the small intestine [122], in accordance with Liao et al.'s data indicating telocyte‐restricted small intestinal expression.

In general, non‐epithelial TSLP expression is becoming increasingly well‐substantiated in mice across a number of tissues, while evidence for TSLP as primarily a lymphoid cell activator is also accumulating. TSLP‐mediated stimulation of ILC2s in the skin [116], intestine [115], and lung [88] has been described, as well as acting on regulatory T cells in the intestine [37, 114, 123], calling into question the relatively poorly evidenced TSLP activity on myeloid cells, and specifically, antigen‐presenting dendritic cells.

## 14 TSLP and Helminth Infection

A mechanism by which TSLP contributes to functional immunity to *Nb* or *Hp* infection has not yet been described: a 2009 study indicated that TSLPR knockout mice mounted normal immune responses and parasite expulsion during *Hp* and *Nb* infection, while *Tm* expulsion and immunity were adversely affected [117]. Excretory‐secretory products from *Hp* and *Nb* were here found to downregulate expression of IL‐12, a cytokine known to act as a pro‐TH1 “signal three” input during antigen presentation, while Tm E/S did not. While this would suggest that TSLP action on dendritic cells is a means for generating type two immunity against Tm infection, DC detection of TSLP was not here demonstrated. Various other studies have replicated a necessity for TSLP signalling in the expulsion of HD *Tm* infection [39, 119], but how it contributes to type two immunity precisely remains poorly characterised. It has been characterised in the small intestine as being telocyte derived, and in other type two anti‐parasite contexts as being a macrophage product: both of these cell types being the most parasite‐adjacent and more abundant non‐epithelial cells in the caecum, they present themselves as the likeliest candidates for upregulation during HD *Tm* infection. The lack of information available regarding how *Tslp* expression is induced in cells impairs in vitro study of cell type potential for TSLP production, making HD *Tm* infection in vivo the most valuable tool presently for furthering understanding of how TSLP contributes to functional immunity.

Potential epithelial production and DC detection of TSLP has been explored [120], but upregulation of *Tslp* by epithelial cells in response to infection, or expression in the caecal epithelium in vivo has not yet been demonstrated; neither has significant expression of the TSLPR by DCs been evidenced. More detailed in vitro work, examining the responsiveness of DCs to TSLP stimulation under a number of conditions, observed increases in CCL17 production, but no other substantial changes [124]. Given numerous instances of TSLP supporting ILC2s and TH2 cells directly in other models [45, 88, 115, 116], direct TSLP licensing of lymphoid effector cell activity or survival is a promising potential avenue for exploring the mechanisms behind its contribution to *Tm* immunity, simultaneously deepening understanding of relevant human diseases, such as asthma.

Alternatively, TSLP induction of basophilia has been explored in more detail as the mechanism on which HD *Tm* immunity relies, albeit with key questions remaining. Basophil Notch signals have been demonstrated to contribute to the expulsion of HD *Tm* [38, 125], and one study has suggested that basophils, in addition to being promoted by TSLP, act as APCs during HD *Tm* infection to promote expulsion [39], although no trafficking of basophils to lymph nodes was observed during HD *Tm* infection, and the mechanisms underpinning basophil presentation of antigen during type two immunity, independent of DCs, have been disputed [126, 127, 128]. TSLP may act to induce IL‐4 producing basophils which provide co‐stimulatory signals during antigen presentation or signalling T cells in the caecum, consistent with Perrigoue et al.'s findings [39]. This is supported by the necessity for basophils in supporting TH2 responses to HD *Tm*, in contrast to IL‐3‐elicited basophilia during Nb infection, which relies on effector T cells already having been activated [129]. TSLP administration results in basophilia [39], and the resulting basophils are transcriptionally distinct from those generated by IL‐3, and demonstrated to be capable of contributing to type two immune responses [130]. Given that IL‐5 and c‐Kit knockouts, on the other hand, largely lacking eosinophils and mast cells respectively, do not experience increased susceptibility to HD *Tm* infection [131], basophils may be uniquely capable of providing key IL‐4 signals necessary for TH2 generation during HD *Tm* infection, but further study on TSLP‐basophil interactions is needed to determine when during infection, where in tissue, and whether TSLP interacts with basophils directly in this potential key signalling event occurs.

B cells, which are influenced by TSLP signals during germinal centre formation [35], remain contentious regarding their connection to HD *Tm* immunity: μMT mice, lacking mature B cells, have been observed as being resistant to a 200 to 300 egg dose [39] but more susceptible to infection with a 175 egg dose than wild types [43], suggesting some contribution by B cells to effective immunity. One study has demonstrated that interruption of follicular helper T cell interactions with B cells results in partial susceptibility to HD *Tm* infection, associated with poor TH2 lymph node responses [44], suggesting that B cells may contribute to immunity by enabling effective TH2 cell responses directly in the mLN. In either case, TSLP may contribute to B cell responses to HD *Tm* as a means of modulating immunity, but whether TSLP production during infection in fact occurs in the lymph node, where B cells would mediate their role, is not clear.

TSLP's demonstrated necessity for HD *Tm* immunity, and the recent emergence of anti‐TSLP treatments for human asthma, make further investigation of the alarmin's presently mysterious mechanism of action particularly valuable. To a greater extent than IL‐33, understanding of TSLP's activity has been greatly shifted by various papers outlining non‐epithelial sources and non‐myeloid recipients for the alarmin.

## 15 Conclusion

**FIGURE 1:**
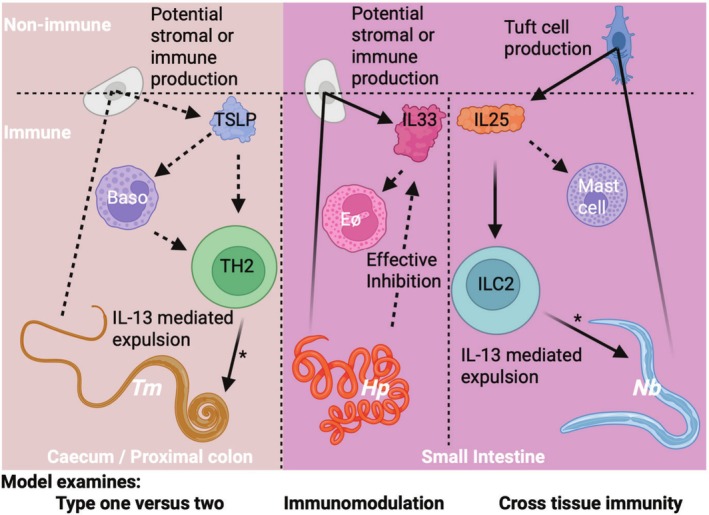
Graphical representation of selected relationships between each classical alarmin and the helminth model most commonly used in its study. Dashed arrows represent evidenced but not mechanistically described interactions; solid arrows represent in vivo demonstrated mechanisms: *represents IL‐13 signalling promoting expulsion via epithelial responses, either directly or indirectly signalling these cell types.

The current model of classical alarmin function in immunology, epithelium‐produced and APC detected, is becoming less supportable: IL‐25 being essentially a tuft cell product, IL‐33 being produced by many cell types within and across tissues, and TSLP being recently characterised as a myeloid and stromal cell product, and all best characterised as functionally signalling lymphoid effector cells as their primary contribution to various helminth infections, with only marginal redundancy between them (Table 1). While epithelium‐derived IL‐33 remains actively investigated alongside non‐epithelial sources, TSLP has essentially been observed as a non‐epithelial cytokine in recent years, despite being commonly stated as an epithelial alarmin. Recent detailed studies support a degree of redundancy between alarmins, but also evidence that they function through different cell types and provoke different and non‐redundant immune responses, useful in different experimental contexts (Figure 1). Dose‐dependent immunity during Tm, cross‐tissue immunity against Nb, and effective immunomodulation by Hp enable different examinations of the immune system, and each has a particularly close relationship with a specific classical alarmin. These studies have largely outlined key roles for alarmins, produced by non‐epithelial sources, acting via lymphoid effector cells and induction of granulocytes to produce anti‐parasite immunity. These new observations create challenges in studying alarmins, but also suggest opportunities for new directions for research, opened by discovering alarmin contributions in unexpected places. While more sub‐cellular examination of IL‐25's activity is ongoing, IL‐33 and TSLP research has been hampered by a lack of clarity regarding their functional sources during helminth infections. IL‐33's activity as a protein has been studied closely, but broad expression and complex interactions with various cell types present issues for relating its function to human diseases. Oppositely, TSLP's connection to human atopic disease has fuelled efforts to develop treatments targeting the cytokine, despite serious gaps in our understanding of its sources or recipients in the intestine or lung. For both cytokines, recent research carefully utilising conditional knockout mice to determine specific relationships between alarmins and recipients in immune models in an unbiased manner [34, 45, 91, 115, 116] has produced surprising answers to fundamental questions around alarmin sources and recipients, foreshadowed by previous work [28]. In this light, researchers may benefit from returning to these basic questions, using new technologies such as spatial transcriptomics to find new niches for alarmins to fill in the ever‐evolving conception of type two immunity [3].

**TABLE 1:** Outlining the contributions of each of the three classical alarmins to expulsion responses against the three most common models of intestinal helminth infection, via their best characterised immune mechanism.

	IL‐25	IL‐33	TSLP
*N. brasiliensis*	Key to 1ry immunity: Tuft cell produced, acts on intestinal ILC2s [32]	Contributor to 2ry immunity [2]; Small intestinal sources contribute conflictingly to expulsion [91], contribution at lung/skin phase unknown.	Minimal impact on immunity [117]
*H. polygyrus bakeri*	Contributes to 1ry and 2ry immunity via unknown cell [33]	Key to 1ry immunity: likely epithelial/stromal/myeloid production [91], suppressed by parasite secretions [9, 10], likely acts on ILC2/TH2 cells [45]	Minimal impact on immunity [117]
*T. muris*	Minimal impact on 1ry immunity [117]	Dispensable for normal HD *Tm* immunity, promotes immunity when administered/upregulated [95]	Key to 1ry immunity to high dose infection: likely stromal/myeloid production [115, 116], acts on BMDSCs/ILC2/TH2 cells [45, 115]

## Conflicts of Interest

The authors declare no conflicts of interest.

## Peer Review

The peer review history for this article is available at https://www.webofscience.com/api/gateway/wos/peer‐review/10.1111/pim.70027.

## Data Availability

Data sharing not applicable to this article as no datasets were generated or analysed during the current study.
